# An Update on Sphingosine-1-Phosphate and Lysophosphatidic Acid Receptor Transcripts in Rodent Olfactory Mucosa

**DOI:** 10.3390/ijms23084343

**Published:** 2022-04-14

**Authors:** JT. Toebbe, Mary Beth Genter

**Affiliations:** Department of Environmental and Public Health Sciences, University of Cincinnati, Cincinnati, OH 45267-0056, USA; toebbejt@mail.uc.edu

**Keywords:** olfactory mucosa, sphingosine-1-phosphate, lysophosphatidic acid, COVID, SARS-CoV-2, receptors

## Abstract

Olfactory neurons connect the external environment and the brain, allowing the translocation of materials from the nasal cavity into the brain. The olfactory system is involved in SARS-CoV-2 infections; early in the pandemic declared in 2020, a loss of the sense of smell was found in many infected patients. Attention has also been focused on the role that the olfactory epithelium appears to play in the entry of the SARS-CoV-2 virus into the brain. Specifically, SARS-CoV-2 enters cells via the angiotensin-converting enzyme 2 protein (ACE2), which is found on supporting cells in the olfactory epithelium. The intranasal administration of sphingosine has been proposed to prevent the binding of SARS-CoV-2 to ACE2. Further, sphingosine-1-phosphate (S1P) receptors appear to facilitate the entry of SARS-CoV-2 into the brain. The goal of these studies was to characterize S1P receptor expression status in rodent olfactory mucosa. The expression of receptors for a related sphingolipid, lysophosphatidic acid (LPA), was also assessed. The results confirm previous reports of S1P1 and S1P3 receptor expression, as well as LPA receptor 1, in mouse olfactory mucosa; moreover, they extend the previous findings to identify additional S1P and LPA receptor transcripts in rat and mouse olfactory mucosa, as well as in cultured olfactory neurons. These findings may enhance the utility of rodent models in identifying agonists and/or antagonists of S1P and LPA receptors that may block the entry of SARS-CoV-2 and other viruses into nasal epithelial cells, and prevent transmission from the nasal cavity into the brain.

## 1. Introduction

SARS-CoV-2 infections clearly affect many organ systems in the human body, including the upper and lower respiratory tracts, central nervous system (CNS), and peripheral nervous system, often with long-term neurological consequences [1]. Neuroimaging studies performed early in the pandemic showed that patients infected with SARS-CoV-2 who displayed neurological symptoms had pronounced central CNS pathology. Lesions included acute infarcts with large clot burdens, and intracranial hemorrhages. Other CNS manifestations of the infection included leukoencephalopathy, global hypoxic injury, acute disseminated encephalomyelitis, cytotoxic lesions of the corpus callosum, cranial nerve involvement, and Guillain–Barré syndrome. Involvement of the olfactory bulb was also noted in these patients [2].

Since the beginning of the COVID-19 pandemic, the olfactory system has been an area of focus. An impaired sense of smell was reported in COVID-19 patients in a multi-center study in Europe, as well as in China [3,4,5]. Relatively early in the pandemic, the US Centers for Disease Control and Prevention added “new loss of taste or smell” to the list of possible indicators of COVID-19 infection [6]. The SARS-CoV-2 coronavirus appears to gain access to the CNS via the olfactory system [7]. Specifically, the virus enters cells via the angiotensin-converting enzyme 2 (ACE2) protein, which is found on many cells in the respiratory tract, including those in the olfactory mucosa [8]. To potentially abate ACE2-dependent entry of SARS-CoV-2 into the CNS, sphingosine nasal sprays have been proposed as a means of preventing the interaction between the SARS-CoV-2 virus and the ACE2 protein [9]. During the response to SARS-CoV-2 infection, immune cells were found to release sphingosine and other sphingolipid metabolites with antimicrobial properties [10]. Similar to the ACE2 protein, sphingosine-1-phosphate (S1P) receptors appear to facilitate entry of the SARS-CoV-2 virus into the nervous system, and the modulation of receptor activity is under investigation in the context of SARS-CoV-2 infection [7,9,10]. S1P is a bioactive lipid involved in immunity, inflammation, angiogenesis, and cancer, and binding to its receptors can modulate inflammatory responses [11]; hence its protective role in the context of SARS-CoV-2 infection may reflect both the restriction of entry of the virus into the CNS, as well as the modulation of CNS inflammation.

Olfactory dysfunction in SARS-CoV-2-infected individuals appears to result from local damage to the olfactory epithelium, as evidenced by histological observations of tissue damage and inflammation. It is possible, though not robustly proven, that the virus may be translocated to the CNS from the infected olfactory epithelium. In a study of post-mortem COVID-19 patients, leukocyte infiltration was found in the lamina propria, with focal atrophy of the olfactory mucosa. There was reportedly limited involvement of the olfactory tracts in these patients [12]. Upon close examination of the olfactory epithelium, ciliated and sustentacular cells were found to contain the virus, while the olfactory sensory neurons and olfactory bulb were not infected [13]. The presence of the SARS-CoV-2 virus was rarely (~6%) detected in the cerebrospinal fluid (CSF) of COVID-19 patients in a study using polymerase chain reaction analysis [14]. In contrast to these observations, olfactory bulb involvement was reported in a larger study examining the brains of post-mortem COVID-19 patients [2], and olfactory nerve damage was reported in another post-mortem case, leading to the conclusion that dissemination from the olfactory epithelium to the CNS is possible [15].

**Figure 1:**
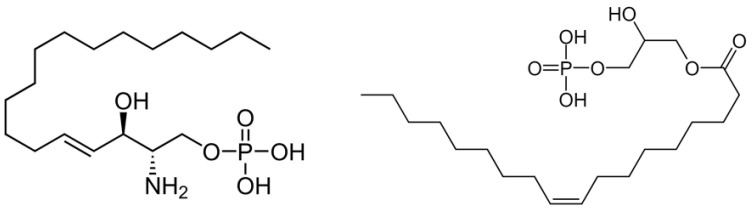
Structures of sphingosine-1-phosphate (**left**) and lysophosphatidic acid (**right**).

A role, or roles, of sphingolipids in the olfactory system was previously proposed, based on findings from a mouse study that was designed to identify highly expressed genes in the olfactory mucosa, as a potential basis for olfactory neuronal plasticity. The findings of the previous study revealed that the sphingosine phosphate lyase gene (*Sgpl1*) was among the most highly expressed genes in the olfactory mucosa, compared to its expression in other brain regions and body tissues [16]. SGPL1 degrades S1P, a sphingolipid catabolite that is involved in calcium signaling and the proliferative signal transduction pathways of many mammalian cells [17,18,19]. SGPL1 was immunohistochemically localized to mature olfactory neurons, and its enzymatic activity was found to be high in both the olfactory mucosa and olfactory bulb of mice [16]. Further, S1P and lysophosphatidic acid (LPA), a closely related lipid signaling molecule, act on families of G-protein-coupled receptors that are implicated in important trophic effects in the nervous system (Figure 1) [20,21,22,23,24]. 

**Figure 2:**
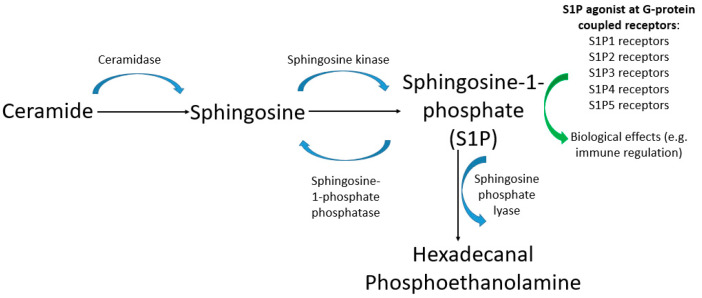
Simplified pathway diagram showing inter-relationship between ceramide, sphingosine, and sphingosine-1-phosphate. Immune responses can include effector T cells entering systemic circulation in response to S1P binding to S1P1 receptors [28].

Olfactory ensheathing cells (OECs), which are a specialized glial cell population with axonal growth-promoting properties in the olfactory system, express multiple S1P receptor transcripts, and are stimulated to proliferate in the presence of S1P. The proliferative response has been noted to require the yes-associated protein (YAP) and proceed through RhoA signaling [25]. Similarly, the Edg-2 (LPA1) receptor was initially described as the first G-protein-coupled receptor to be found to be selectively expressed in myelin-forming cells in the nervous system. Further, its temporal expression pattern was regarded as being consistent with a dual role for LPA signaling in neurogenesis during embryonic development, and in myelination during postnatal life [26]. More recently, OECs have been found to express LPA1, LPA2, and LPA3 receptor transcripts. These studies demonstrated that when OECs were treated with LPA in vitro, both proliferation and migration of OECs was observed, as were actin cytoskeleton reorganization and focal adhesion assembly via a pathway involving Rho-GTPase, MAPK, and PI 3-K signaling cascades [27]. A schematic representation of sphingolipid signaling is presented in Figure 2.

S1P and LPA bind to a family of G-protein-coupled receptors; these were originally called the endothelial differentiation gene (EDG) receptor family [29], but are now referred to as S1P and LPA receptors, with five and four members, respectively [20]. The complement of EDG/S1P receptors expressed by different cell types and tissues appears to correlate with the mitogenic effects of exogenously administered S1P. For example, S1P is mitogenic for striatal astrocytes [30] and endothelial cells [31], both of which express EDG1 (S1P1), EDG3 (S1P3), but not EDG5 (S1P2) receptors. In contrast, cells that express EDG8 (S1P5) appear to be inhibited in a proliferative capacity by S1P [32]. The results of an in situ hybridization study suggested that the S1P1 receptor subclass is associated with embryonic neurogenesis and angiogenesis in mice [33]. Studies on both the distribution of the receptors and the effects of gene knockout demonstrate that almost all receptors play important roles in the survival and differentiation of nervous system structures. For example, the LPA1 receptor is highly expressed in the cerebral cortex and olfactory bulb of embryonic mice. Mice null for the LPA1 receptor showed a high degree of mortality at birth, and survivors displayed decreased sucking behavior and stunted growth, which is consistent with olfactory dysfunction [34]. Nevertheless, the expression pattern of other S1P and LPA receptors in many tissues is not currently fully understood. 

Several S1P and LPA receptor transcripts have previously been described in mouse olfactory mucosa. For example, after the isolation of olfactory epithelial cells from mice expressing green fluorescent protein, under the control of the Neurog1 promoter to fluorescently label cells of an olfactory neuronal lineage, S1P1 and S1P3 receptor transcripts were primarily observed in non-neuronal structures and mature olfactory sensory neurons (OSNs), while S1P3 receptor transcripts were comparatively less expressed in immature OSNs [35]. Similarly, the LPA1 receptor transcript was found predominantly in mature olfactory neurons and non-neuronal structures [35]. A proteomic analysis supported this initial observation by demonstrating enrichment of LPA1 receptor expression in olfactory sensory cell cilia [36]. 

Therefore, the goal of our studies was to complement previous S1P and LPA receptor expression observations in olfactory tissue, by elucidating the expression status of various other S1P and LPA receptor transcripts in rat and mouse olfactory mucosa, as well as in the rat olfactory neuronal cell line Odora [37]. Findings from this study may enhance the utility of rodent models in identifying agonists and/or antagonists of S1P and LPA receptors that may have beneficial health effects, such as blocking the entry of SARS-CoV-2 and other viruses from the nasal cavity into the CNS, and decreasing neuroinflammation.

## 2. Results and Discussion

As summarized in Table 1, rat and mouse olfactory tissues, as well as a rat olfactory neuronal cell line (Odora cells [37]) express multiple mRNA transcripts for S1P and LPA receptors. Both rat and mouse olfactory mucosal tissues expressed S1P1, 2, 3, and 4, but not S1P5 receptors. Mouse tissues expressed LPA1, 2 and 3 receptors, while rat tissues expressed LPA1 and LPA2 receptors, but not LPA3 receptors. In the Odora cell line, differentiated cells (corresponding to mature olfactory neurons in vivo) expressed S1P1, 2, 3, and 4 receptors, as well as the LPA1 receptor, but not the LPA2 or 3 receptors. Undifferentiated Odora cells, corresponding to immature olfactory neurons in vivo, additionally expressed S1P4. While receptor expression in RNA isolated from the intact olfactory mucosa does not specify the cell type expressing the receptors, the receptor profile of Odora cells identifies S1P and LPA receptors, namely S1P1, 2, 3 and 4 and LPA1 receptors, on the mature olfactory neurons themselves, given that Odora cells are derived from olfactory receptor neurons [37]. 

Thus, the new knowledge gained in this study includes the expression of S1P2, S1P4, and LPA2 receptors on both rat and mouse olfactory mucosa, as well as the LPA3 receptor on mouse olfactory mucosa. The identification of these new transcripts may have important clinical implications. For example, in chronic obstructive lung disease and sepsis, a link is proposed between S1P2 signaling and diminished phagocytic function [38,39]. Further, another study demonstrated that JTE-013, an inhibitor of S1PR2, decreased the replication of human cytomegalovirus [40]. Therefore, agents that block signaling through the S1P2 receptor as inhibitors or antagonists, or agents that cause receptor internalization, might have beneficial effects in certain viral infections. Whether these observations extend to SARS-CoV-2 is to be determined.

Ohuchi et al. [41] performed a total-body evaluation of LPA and S1P receptors in E12.5 mouse embryos. The expression of LPA1, 2, and 4 receptors was detected in the head/face region, while the nasal region displayed the expression of LPA1-4 receptors, as well as S1P2 and S1P3 receptors. In terms of LPA receptor expression in the developing mouse brain, Hecht and collaborators found that the LPA1 receptor is expressed in the ventricular zone of the rat embryonic brain; the timing and location of the expression suggest that it is expressed by dividing neuronal stem/progenitor cells [42]. This is particularly important in light of the migration of adult-derived neurons from the subventricular zone to the olfactory bulb, as well as ongoing neurogenesis in the olfactory mucosa throughout life. LPA1 receptor knockout mice displayed perinatal lethality and deficits in the proliferation of neuronal progenitor cells [43]. The LPA1 receptor is also implicated in olfaction. In an LPA1 knockout mouse model, neonatal pups showed impaired suckling, leading to 50% neonatal lethality; this was proposed to be due to defective olfaction, perhaps due to developmental abnormalities in the olfactory bulb or other central components of olfactory processing [34]. While our studies did not detect S1P5 receptor expression in cDNA derived from olfactory mucosa, mouse OECs—which are glial cells found in close contact with olfactory axons in the olfactory nerve and olfactory bulb, and are also currently under study for the repair of spinal cord injuries—reportedly did express S1P5 [25,44]; this demonstrates some compartmentalization of receptor expression in different olfactory structures.

A role for S1P receptors in viral infections has previously been examined in mouse models of the H1N1 influenza virus infection [45,46]. These studies focused on infections of lung epithelial cells. Following instillation of the H1N1 virus into the lung, mice who were administered the S1P receptor agonist AAL-R via intratracheal instillation were protected from a cytokine storm, and hence, from severe lung injury. The beneficial effect was attributed to the modulation of both dendritic and CD8-positive T cell function [45]. Another S1P receptor agonist, CYM-5442, provided a protective effect in the context of H1N1 infection [46]. These investigators also demonstrated that the absence of the S1P1 receptor on endothelial cells enhanced immune-mediated pulmonary injury in the mouse model of H1N1, and that sphingolipid signaling did not affect viral clearance in mice infected with H1N1 [46]. Fingolimod (FTY720), the oldest sphingosine analogue approved for the treatment of multiple sclerosis (MS), is also under evaluation for clinical intervention in SARS-CoV-2 infections [47]. Fingolimod acts as a selective functional antagonist of the S1P1 receptor subtype by inducing receptor downregulation, leading to immunosuppression. Tasat and Yakisich [47] reviewed studies of MS patients undergoing treatment with FTY720, most of whom had mild or no symptoms of COVID-19 disease, despite being found to be infected with the SARS-CoV-2 virus. Therefore, with the knowledge of additional S1P and LPA receptor expression in rodent olfactory mucosa, the pathways modulated by these receptors may be exploited in the recovery of olfaction or the delivery of S1P receptor agonists and/or antagonists to the brain. For example, antagonism of S1P3 receptors in rats in vivo with CAY10444 was associated with neuroprotection [48] in a model of acute intracerebral hemorrhage. Our results provide novel data on S1P4 receptor expression in adult rodent olfactory mucosa, with expression likely on olfactory neurons based on S1P4 receptor expression; these are also found on Odora cells, which are derived from rat olfactory neurons [37] (Table 1). As another example of novel benefits associated with S1P receptor modulation, S1P4 receptor manipulation using knockout mice, S1P4 receptor siRNA, as well as pharmacological agonists and antagonists of S1P4 receptors, revealed a positive role for S1P4 receptor in maintaining blood–brain barrier (BBB) integrity, suggesting that the S1P4 receptor may be a drug target in CNS diseases associated with BBB dysfunction [49].

In summary, the COVID-19 pandemic renewed interest in the olfactory system, based on the loss of olfactory function in COVID-19 patients and neuroinflammation that is attributed, in part, to the ability of the SARS-CoV-2 virus to enter the CNS via the olfactory mucosa [4,5,6,50]. Sphingolipid receptors and their signaling in the olfactory system have also come to the forefront, based on the potential of sphingosine to prevent SARS-CoV-2 from binding to ACE2 and entering the nasal epithelial cells, and eventually the CNS [9]. Knowledge of the S1P and LPA receptor transcripts present on OECs is being utilized to understand brain development and to develop potential treatments for serious conditions, such as spinal cord injuries and hemorrhagic brain lesions [27,44]. Therefore, new knowledge about S1P and LPA expression on the olfactory mucosa and olfactory neurons may give some clues about the unique neuroplasticity of olfactory neurons, and may also provide other therapeutic opportunities. Given the potential for long-term sequelae of SARS-CoV-2 infections of the nervous system [1], further investigations in rodent models might uncover an important role for other agonists and antagonists of these receptors, administered intranasally, in preventing the spread of SARS-CoV-2, as well as other potential viruses, from the nasal cavity to the brain. 

## 3. Materials and Methods

Animal studies were approved by the University of Cincinnati Institutional Animal Care and Use Committee, and were designed for minimal animal distress. Odora cells were grown under differentiated conditions (four days in differentiation medium at 39 °C) and under undifferentiated conditions (in growth medium at 33 °C; culture conditions were used as previously described [37]). Total RNA was isolated from Odora cells grown under differentiated and undifferentiated conditions, as well as the olfactory mucosa derived from two male 10-week-old Long Evans rats and two male 8-week-old C57BL/6J mice after euthanasia with carbon dioxide, using TRI Reagent (MRC, Cincinnati, OH, USA) per the manufacturer’s directions. One-microgram aliquots of DNAse-treated RNA were reverse transcribed using the AbGene kit (Thermo Fisher Scientific, Waltham, MA, USA). S1P and LPA receptor expression was queried using published primer pairs for the rat [51] and mouse [52,53]. Primers not found in the published literature were designed based on published nucleotide sequences. Primer sequences are detailed in Table 2. PCR conditions for rat primers were as follows: 95 °C for 3 min, followed by 35 cycles of 95 °C for 30 s; 52 °C for 1 min; 72 °C for 1 min; and a final extension of 10 min at 72 °C. For rat LPA2, an annealing temperature of 62 °C was used. PCR conditions for mouse primers were as follows: 95 °C for 3 min, followed by 40 cycles of 95 °C for 30 s; 55 °C for 1 min; 72 °C for 1 min; and a final extension of 10 min at 72 °C. PCR products were electrophoresed through agarose gels, and amplicon size was determined using a DNA ladder (GeneRuler, Thermo-Fisher Scientific, Waltham, MA, USA 100 bp) and ethidium bromide staining.

## Figures and Tables

**Table 1:** S1P and LPA receptors detected in rat and mouse olfactory mucosa (OM) by RT-PCR.

Current Name	Alternate Name	Presence in Rat OM Detected by RT-PCR	Presence in Mouse OM Detected by RT-PCR	Presence in Differentiated Odora Cells
S1P1	EDG-1	+	+	+
S1P2	EDG-5	+	+	+
S1P3	EDG-3	+	+	+
S1P4	EDG-6	+	+	++
S1P5	EDG-8	--	--	--
LPA1	EDG-2	+	+	+
LPA2	EDG-4	+	+	--
LPA3	EDG-7	--	+	--

+ Presence detected by RT-PCR; ++ also present in undifferentiated Odora cells; -- not detected by RT-PCR.

**Table 2:** Primer sequences for RT-PCR analysis of rat and mouse S1P and LPA receptors in rat and mouse olfactory mucosa and rat-derived Odora cells.

Current Name	Alternate Name	Forward Primer (Rat) * 5′ -> 3′	Reverse Primer (Rat) 5′ -> 3′
S1P1	EDG-1	CTTCAGCCTCCTTGCTATCG-	GCAGGCAATGAAGACGACACTCA
S1P2	EDG-5	TTCTGGTGCTAATCGCAGTG	GAGCAGAGAGTTGAGGGTGG
S1P3	EDG-3	TCAGGGAGGGCAGTATGTTC+	CTGACTCTTGAAGAGGATGG+
S1P4	EDG-6	GTGCTCAACTCAGCCATCAA	CTGCCAAACATTCATCATGG
S1P5	EDG-8	TGTTCCTGCTCCTGGGTAGT	GTTTCGGTTGGTGAAGGTGT
LPA1	EDG-2	ATTTCACAGCCCCAGTTCAC	ACAATAAAGGCACCCAGCAC
LPA2	EDG-4 ^#^	GGCCTACCTCTTCCTCATGTT	GCACATAGAAGAAAATTCGTG
LPA3	EDG-7	TGAGCCTCCATGTGTAGCTG	AGCTTGTGCAGCCTCTCTTC
		Forward primer (mouse) **, *** 5′ -> 3′	Reverse primer (mouse) 5′ -> 3′
S1P1	EDG-1	CACCGGCCCATGTACTATTT	GACTGCCCTTGGCGATGTTC
S1P2	EDG-5	GGGCATGTCACTCTGTCCTT	GACGGGACAAGGTGGAGTCTA
S1P3	EDG-3 ^##^	ATGGCAACCACGCATGCGCA	CAATGATGCAGGAAGAAGTA
S1P4	EDG-6	GGCTACTGGCAGCTATCCTG	GCTGAGTGACCGAGAAGTCC
S1P5	EDG-8	GCCGGTGAGTGAGGTTATTG	CGCGACATCCAGTAATAGCA
LPA1	EDG-2	GAGGAATCGGGACACCATGAT	ACATCCAGCAATAACAAGACCAATC
LPA2	EDG-4	GGCCTACCTCTTCCTCATGTT	GCACATAGAAGAAAATTCGTG
LPA3	EDG-7	GCTCCCATGAAGCTAATGAACACA	AGGCCGTCCAGCAGCGA

* Sequences from Hornuß et al. [51], except where noted; ** LPA sequences from Hama et al. [52]; *** S1P sequences from Whetzel et al. [53] except where noted; ^#^ LPA2 sequence from Lee et al. [54]; ^##^ designed in-house using NCBI sequence data.

## Data Availability

Not applicable.
